# A Longitudinal Case Study of Recurrent Pyostomatitis-Pyodermatitis Vegetans Followed by the Subsequent Development of Bullous Pemphigoid With the Presence of Anti-BP230 Autoantibodies Six Years Later

**DOI:** 10.7759/cureus.88865

**Published:** 2025-07-27

**Authors:** Qian Ren, Yao Liu, Jiaqi Wang, Shuang Ren, Xiaobing Guan

**Affiliations:** 1 Department of Oral Medicine, Beijing Stomatological Hospital, Capital Medical University, Beijing, CHN

**Keywords:** anti-bp230 antibodies, bullous pemphigoid, pyodermatitis vegetans, pyostomatitis vegetans, ulcerative colitis

## Abstract

Pyostomatitis vegetans (PSV) is a rare inflammatory condition that has been strongly associated with inflammatory bowel disease (IBD). Additionally, certain cases of PSV have been reported to co-occur with pyodermatitis vegetans (PDV), resulting in a distinct mucocutaneous manifestation. Herein, we present a clinical case of PSV in a 64-year-old male patient diagnosed with ulcerative colitis (UC). The oral lesions demonstrated complete resolution following treatment with systemic glucocorticoids and immunosuppressants, in conjunction with targeted therapy for the underlying intestinal disease. During longitudinal follow-up, the patient subsequently developed PDV and, unexpectedly, exhibited recurrent bullous eruptions on cutaneous surfaces, specifically localized to the axillary and dorsal regions six years post-initial diagnosis. Enzyme-linked immunosorbent assay (ELISA) analysis revealed significantly elevated titers of circulating anti-BP230 antibodies in the serum samples. These clinical and immunologic findings, together with histopathological evaluation of the cutaneous lesions, established an additional diagnosis of bullous pemphigoid (BP). Both BP and PSV-PDV conditions remained clinically stable under treatment with topical and systemic glucocorticoids, with circulating anti-BP230 antibody titers normalizing eight months after the initiation of therapy. To the best of our knowledge, this represents the first documented case of a patient with PSV-PDV developing BP mediated by anti-BP230 antibodies during long-term follow-up observation. This finding underscores a potential novel disease association that warrants further exploration.

## Introduction

Pyostomatitis vegetans (PSV) is a rare, chronic inflammatory condition affecting the oral mucosa, with its underlying etiopathogenesis remaining largely elusive. A well-established association exists between PSV and inflammatory bowel disease (IBD), particularly ulcerative colitis (UC), as extensively documented in the literature [1]. The clinical manifestations of PSV are characterized by the presence of multiple erythematous pustules, which may rupture and lead to extensive erosions. Additionally, certain oral lesions may display distinct linear patterns resembling the "snail track" morphology [2]. The cutaneous manifestations of this condition are clinically designated as pyodermatitis vegetans (PDV). Peripheral eosinophilia is commonly observed in patients with PSV-PDV. Histopathological analysis of PSV-PDV demonstrates prominent epithelial hyperplasia associated with dense mixed inflammatory cell infiltrates, as well as intraepithelial or subepithelial eosinophilic and neutrophilic microabscesses. When acantholysis is present, it is typically focal and mild [3]. Direct immunofluorescence (DIF) findings are typically negative. However, in specific instances, DIF results may reveal immunoreactivity patterns that are analogous to those observed in autoimmune bullous diseases, with faint deposits of IgG, C3, and IgA along the basement membrane zone or on epithelial cell surfaces [4,5]. Furthermore, circulating antibodies against BP180 or BP230 have occasionally been detected in patient sera during the active phase of PSV-PDV, as reported in some case studies [6,7]. These autoantibodies have been firmly established as critical factors driving the pathogenesis of bullous pemphigoid (BP), where they mediate tissue damage and contribute to the development of bullous eruptions [8]. The identification of these autoantibodies implies a potential overlap between PSV-PDV and autoimmune bullous diseases, suggesting a common immunopathogenic mechanism. Herein, we present a notable case of PSV-PDV in which high-titer anti-BP230 antibodies were detected six years after the initial clinical remission, thereby resulting in the onset of BP.

## Case presentation

A 64-year-old male patient was referred to the Department of Oral Medicine in August 2018 for the evaluation and management of multiple painful oral ulcerations that had persisted for one month. His medical history includes a three-year course of UC with occasional relapses. Concurrently with the development of oral ulcers, the patient experienced a recurrence of increased stool frequency accompanied by the presence of pus and blood-tinged mucus in the stool over the past several days. Based on the clinical presentation and colonoscopic findings, a diagnosis of recurrent UC was established. The patient was treated with mesalazine enteric-coated tablets (1.0 g per dose, three times daily, orally) along with traditional Chinese medicine enema therapy. Consequently, gastrointestinal symptoms showed observable improvement; however, oral ulcers progressively worsened. The patient reported no prior history of cutaneous lesions or involvement of other mucosal surfaces, including ocular, genital, and nasal mucosae.

**Figure 1:**
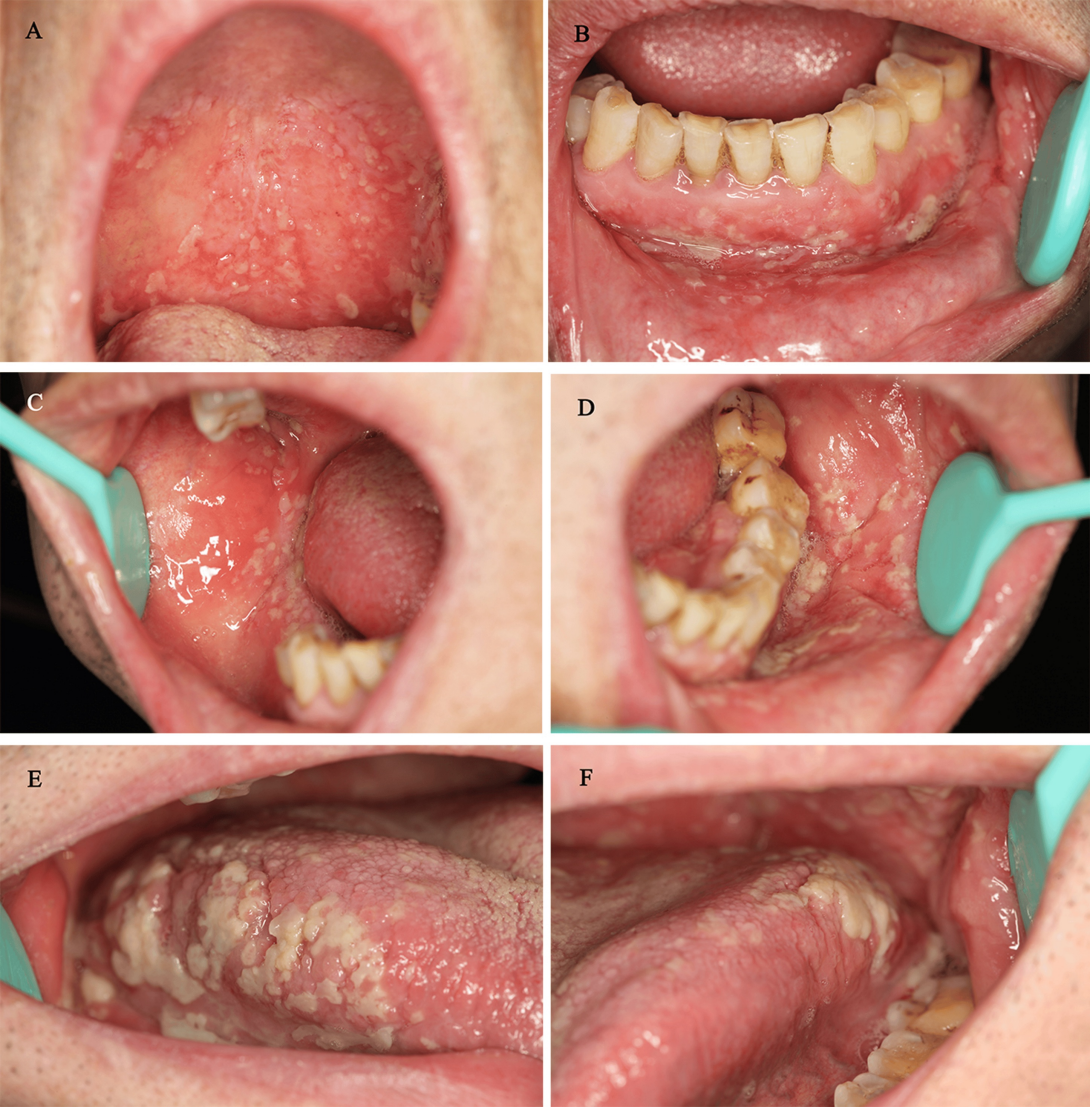
Oral manifestations observed at the initial presentation (A) Multiple exophytic erosions merged to form “snail-track” appearances on the soft palate. (B-F) Widespread pustular erosions on alveolar gingivae, buccal mucosa, and tongue

**Figure 2:**
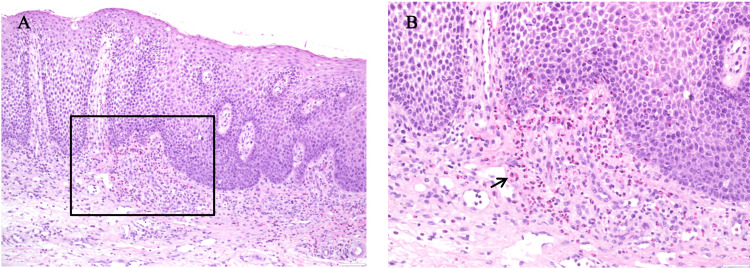
Histopathological findings of erosions on the lower lip mucosa H&E: hematoxylin and eosin (A) Epidermal hyperplasia with mixed inflammatory cell infiltrate within the lamina propria (H&E stain, original magnification ×100). (B) Abundant eosinophils in the lamina propria (arrow) (H&E stain, original magnification ×200)

Clinical examination revealed multiple small exophytic erosions with erythematous borders and creamy white to yellow pseudomembranous surfaces. These lesions extensively affected the soft palate, maxillary and mandibular alveolar gingivae, buccal mucosa, and tongue. Some of the shallow erosions coalesced, forming linear “snail-track” appearances on the soft palate (Figure 1). Nikolsky's sign was negative upon evaluation of these lesions. Laboratory testing demonstrated marked peripheral eosinophilia, with an eosinophil percentage of 17.9% (reference range: 0.4% to 8.0%) and an absolute eosinophil count of 1.50 × 10^9^/L (reference range: 0.02 × 10^9^/L to 0.52 × 10^9^/L). Histopathological examination of the lower lip mucosa revealed prominent epidermal hyperplasia along with a dense, mixed inflammatory cell infiltrate in the lamina propria. The infiltrate was predominantly composed of eosinophils, interspersed with lymphocytes and plasma cells. Notably, neither acantholysis nor intraepithelial splitting was observed (Figure 2). Serological testing performed via enzyme-linked immunosorbent assay (ELISA) showed negative results for anti-Dsg1 antibodies (1.71 RU/ml, normal range<20 RU/ml),anti-Dsg3 antibodies (8.09 RU/ml, normal range <20 RU/ml) and anti-BP180 antibodies (0.39 RU/ml, normal range <20 RU/ml). A definitive diagnosis of PSV was established based on an integration of these clinical evaluations, laboratory findings, and a thorough review of the patient's medical history and profile.

Treatment and outcome

The therapeutic regimen consisted of systemic oral prednisone, initiated at a dose of 30 mg/day, supplemented by topical application of 0.1% triamcinolone acetonide dental paste, which was administered twice daily for the effective management of oral lesions. Following one week of treatment, all oral lesions had completely resolved, and the prednisone dosage was systematically tapered according to clinical protocol. However, during the tapering of corticosteroids, recurrent small pustular erosions appeared on the oral mucosa. This led to the initiation of thalidomide (50 mg/day) as an adjunctive therapy for a period of seven months to help prevent relapse. Longitudinal surveillance exhibited a gradual and sustained improvement in oral lesions throughout the subsequent monitoring period. At the eight-month follow-up examination, the oral mucosa appeared normal, with no significant abnormalities. Concomitantly, prednisone had been successfully tapered to a maintenance dose of 10 mg/day.

Follow-up

Following the completion of the initial treatment phase, the patient did not adhere to the scheduled regular follow-up appointments. Over the subsequent five-year period, the patient experienced recurrent oral erosions occurring biannually, with each episode persisting for approximately three months. Notably, since 2021, he has concurrently developed cutaneous erythematous erosions in addition to oral lesions. These mucocutaneous manifestations have been consistently correlated with exacerbations of UC. The patient adopted a self-regulated methylprednisolone dosing regimen (8-16 mg/day) to maintain a sustained clinical response. Nevertheless, when the corticosteroid dosage was decreased below 6 mg/day, mucocutaneous symptoms always recurred, thereby requiring long-term administration of methylprednisolone to maintain remission.

**Figure 3:**
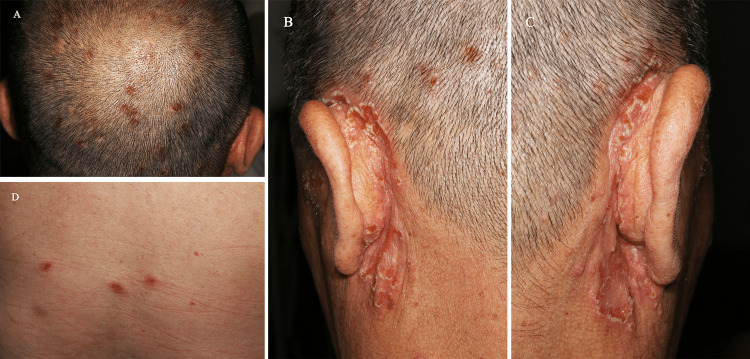
Distinctive cutaneous manifestations observed at the six-year follow-up (A-C) Well-defined pustular erosions with annular pseudomembranes on the scalp and ear. (D) Post-bullous erosions on the dorsal skin

**Figure 4:**
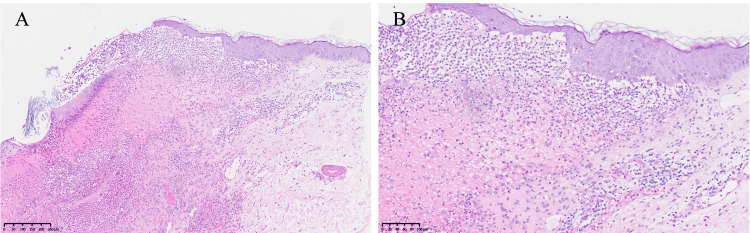
Histopathological features of post-bullous erosions on the dorsal skin (A-B) Subepidermal fissures with mixed inflammatory cell infiltrate in the superficial to mid-dermis. (H&E stain, (A) original magnification ×100; (B) original magnification ×200)

In May 2024, the patient reported a disease recurrence characterized by extensive mucocutaneous lesions. Unlike previous disease flares, the current relapse presented with a clinically distinct vesiculobullous cutaneous phenotype. Multiple tense and intact bullae were observed in the bilateral axillary and dorsal regions, exhibiting resistance to rupture. At the time of referral to our department, the patient had been experiencing recurrent bullous eruptions that had persisted for about two months. On physical examination, multiple erythematous lesions were identified on the scalp and posterior auricular regions. These lesions had a pustular quality and displayed superficial erosions covered with annular yellow pseudomembranes or crusts, which were reminiscent of those observed in PSV lesions of the oral mucosa (Figures 3A-3C). In contrast to the aforementioned primary cutaneous manifestations, the dorsal skin exhibited three well-demarcated erosive regions consequent to bullae rupture. These areas were covered with hemorrhagic crusting and encircled by erythematous margins (Figure 3D). Furthermore, the oral lesions remained consistent with the previously documented clinical presentations of PSV. Histopathological examination of the dorsal skin lesion showed subepidermal fissures, accompanied by perivascular infiltration predominantly composed of neutrophils and a scattered presence of eosinophils in the superficial to mid-dermis. Focal thrombi were observed within certain dermal vessels (Figure 4).

The ELISA assay for anti-BP antigen 230 (BP230) antibodies demonstrated a positive result (136.66 RU/ml; normal range <20 RU/ml), whereas assays for anti-BP180, anti-Dsg1, and anti-Dsg3 antibodies were all negative. Based on these findings, the dermatologist established a dual diagnosis of PDV and BP. Methylprednisolone treatment was initiated at a dose of 40 mg/day, and a marked improvement was noted following the commencement of therapy. Eight months later, the titer of anti-BP230 antibodies decreased to 16.76 RU/ml (normal range <20 RU/ml), and the patient has remained asymptomatic to the present time.

## Discussion

The diagnosis of PSV, a rare oral manifestation associated with inflammatory bowel disease, was confirmed upon the patient's initial presentation based on the following key clinical and laboratory findings: (1) extensive pustular erosions on the oral mucosa with characteristic "snail-track" patterns on the palate; (2) peripheral eosinophilia identified in complete blood count; (3) histopathological examination revealing prominent eosinophilic infiltration within the subepithelial layer; and (4) a concurrent exacerbation of pre-existing UC temporally correlated with the onset of oral lesions. PSV must be primarily differentiated from pemphigus vegetans (Hallopeau type) [9]. In clinical practice, the precise differentiation between these two conditions largely depends on DIF findings and the detection of circulating anti-desmoglein antibodies [10]. Although DIF analysis was not performed during the initial tissue biopsy, the integration of the aforementioned four diagnostic criteria, along with the negative serological results for anti-Dsg1 and anti-Dsg3 antibodies, allowed us to reliably exclude pemphigus vegetans (Hallopeau type). Moreover, the patient's initial clinical and pathological features were markedly distinct from those observed in mucous membrane pemphigoid and BP, thereby effectively ruling out these subepidermal blistering disorders.

At the six-year follow-up examination, multiple pustular erosions surrounded by annular pseudomembranes were observed on the scalp and bilateral retroauricular skin, exhibiting pathognomonic features characteristic of PDV [11]. Notably, the patient had experienced recurrent episodes of bullous eruptions over the past two months, primarily involving the axillary and dorsal regions. Dermatological assessment revealed three discrete erosive lesions on the back, resulting from the rupture of previously existing bullae. The combination of recurrent bullous eruptions, post-bullous erosions on the dorsal skin, histopathological confirmation of subepidermal fissures, and the detection of high-titer anti-BP230 antibodies collectively substantiates the definitive diagnosis of BP.

The immunological mechanisms responsible for the production of anti-BP230 antibodies in this patient with PSV-PDV remain poorly understood. The precise timing of the onset of anti-BP230 antibody production could not be ascertained, as serological testing for these autoantibodies was not performed routinely at the time of the patient’s initial presentation. A distinct case of PSV-PDV with concomitant anti-BP230 antibodies has previously been reported, in which the author interpreted this serological observation as an epiphenomenon potentially resulting from inflammation-mediated epidermal damage in PSV-PDV [7]. Moreover, in a specific case of PSV-PDV, circulating anti-BP180 antibodies were detected during the active phase of the disease, but became undetectable during the remission phase. The possible mechanism underlying the presence of these antibodies may be secondary to the exposure or unmasking of basement membrane zone (BMZ) antigens [6]. It is noteworthy that, although both patients demonstrated seropositivity for BP-specific autoantibodies (anti-BP230 or anti-BP180) in the two documented cases, neither exhibited typical clinical manifestations of BP. As integral structural components of the hemidesmosome adhesion complex, both BP230 and BP180 function as primary autoantigenic targets in BP. In addition, autoantibodies specifically targeting either BP230 or BP180 individually exhibit pathogenic potential in inducing subepidermal bullae, which are a hallmark clinical feature of BP, as evidenced by both clinical observations and experimental models [12-14]. We can conclude that patients with PSV-PDV who exhibit circulating anti-BP180 or BP230 antibodies appear to be at a significantly higher risk of developing BP, highlighting the critical need for long-term clinical monitoring in this population. The current clinical case presents preliminary evidence for a novel pathogenic connection between PSV-PDV and BP. We hypothesize that PSV-PDV-associated inflammation may act as a credible pathophysiological driver for BMZ disruption. Such a sustained inflammatory state may potentially expose previously masked antigens, thereby triggering an autoimmune response directed against BMZ components, including BP230. This process may ultimately lead to the synthesis of anti-BP230 autoantibodies and eventually contribute to the development of BP-related cutaneous manifestations. Validation of this hypothesis necessitates a systematic evaluation of comparable PSV-PDV cases with longitudinal immunological monitoring and experimental verification.

## Conclusions

This case represents a clinically significant instance of recurrent PSV-PDV, subsequently complicated by the development of BP, as evidenced by the presence of high-titer anti-BP230 autoantibodies. This finding underscores the imperative for long-term clinical follow-up and comprehensive immunological monitoring among PSV-PDV patients, with an emphasis on meticulous lesion evaluation and early detection of disease-specific biomarkers. Routine screening for pemphigoid antibodies, encompassing both anti-BP180 and anti-BP230 antibodies, is strongly recommended as an essential component of the monitoring protocol. Furthermore, this case demonstrates the critical importance of a multidisciplinary approach in enhancing patient care. Achieving effective management for this patient necessitated collaborative efforts across multiple specialties, such as gastroenterology, oral medicine, and dermatology, to achieve sustained clinical remission and optimal therapeutic outcomes.
